# Supplementary material for: The Effects of Imagery Practice on Athletes’ Performance: A Multilevel Meta-Analysis with Systematic Review
Source: Behav Sci (Basel). 2025 May 16;15(5):685. doi: 10.3390/bs15050685 (PMC12109254; doi:10.3390/bs15050685)
Supplement: Supplementary file 1 [file behavsci-15-00685-s001.zip › Supplementary file S1 Search strategy.pdf]

Supplementary File S1 : Search Strategy

EBSCO (MEDLINE complete, MEDLINE, CINAHL, PsycInfo, SPORTDiscus)<br>Febrary 22 2025	
1	TI(Imagery* OR “Imagery practice”)
2	AB(Imagery* OR “Imagery practice”)
3	TI(Imaging*)
4	AB(Imaging*)
5	1 OR 2 OR 3 OR 4
6	TX(exerci* OR exercise* OR “athletic performance” OR athletic* OR sports* OR sport* OR “sports performance” OR strength* OR “muscle strength” OR “endurance performance” OR endurance* OR agility* OR “cognitive ability” OR “response time” OR aerobic* OR anaerobic*)
7	TX(athletes* OR players* OR “para-athletes” OR cyclists* OR runners* OR golfers* OR wrestlers* OR “rugby players” OR “tennis players” OR boxers* OR “disabled athletes” OR shooters*)
8	5 AND 6 AND 7

	Pubmed<br>Febrary 22 2025
1	(((Imagery[MeSH Terms]) OR (Imagery[Title/Abstract])) OR (Imaging[Title/Abstract])) OR ("Imagery practice"[Title/Abstract])
2	((((((((((athletic performance[MeSH Terms]) OR (sport performance[Text Word])) OR (sports performance[Text Word])) OR (athletic performance[Text Word])) OR (sport[Text Word])) OR (athletic[Text Word])) OR (endurance performance[Text Word])) OR (performance[Text Word])) OR (strength[Text Word])) OR (power[Text Word])) OR (anaerobic[Text Word])) OR (aerobic[Text Word])
3	(((((((((((((athletes[MeSH Terms]) OR (athletes[Title/Abstract])) OR (athletes[Text Word])) OR (players[Text Word])) OR (players[Title/Abstract])) OR (runners[Text Word])) OR (cyclists[Text Word])) OR (boxers[Text Word])) OR (shooters[Text Word])) OR (wrestlers[Text Word])) OR (golfers[Text Word])) OR ("para-athletes"[Text Word])) OR ("rugby players"[Text Word])) OR ("tennis players"[Text Word])
4	1 AND 2 AND 3

Web of Science<br>Febrary 22 2025	
1	((TS=(Imagery* OR Image* OR "Imagery practice" OR Imaging* OR "Imagery training")) OR TI=(Imagery* OR Image* OR "Imagery practice" OR Imaging* OR "Imagery training")) OR AB=(Imagery* OR Image* OR "Imagery practice" OR Imaging* OR "Imagery training")
2	ALL=(exerci* OR exercise* OR “athletic performance” OR athletic* OR sports* OR sport* OR “sports performance” OR strength* OR “muscle strength” OR “endurance performance” OR endurance* OR agility* OR “cognitive ability” OR “response time” OR aerobic* OR anaerobic*)
3	((ALL=(athletes* OR players* OR “para-athletes” OR cyclists* OR runners* OR golfers* OR wrestlers* OR “rugby players” OR “tennis players” OR boxers* OR “disabled athletes” OR shooters*)) OR TI=(athletes* OR players* OR “para-athletes” OR cyclists* OR runners* OR golfers* OR wrestlers* OR “rugby players” OR “tennis players” OR boxers* OR “disabled athletes” OR shooters*)) OR AB=(athletes* OR players* OR “para-athletes” OR cyclists* OR runners* OR golfers* OR wrestlers* OR “rugby players” OR “tennis players” OR boxers* OR “disabled athletes” OR shooters*)
4	#1 AND #2 AND #3
