# Supplementary material for: The Effects of Imagery Practice on Athletes’ Performance: A Multilevel Meta-Analysis with Systematic Review
Source: Behav Sci (Basel). 2025 May 16;15(5):685. doi: 10.3390/bs15050685 (PMC12109254; doi:10.3390/bs15050685)
Supplement: Supplementary file 1 [file behavsci-15-00685-s001.zip › Supplementary file S3 Risk_of_Bias_in_Individual_Studies.pdf]

Table 1: <i> Supplement File. Risk of Bias in Individual Studies </i>

Author	Sequence generation	Allocation concealment	Blinding participants	Blinding personnel	Blinding outcome assessment	Incomplete outcome	Selective reporting	Other bias	Overall bias
Abraham	Low	Low	Low	Low	Unclear	Low	Low	Low	Low
Amini Farsani	Low	Unclear	Unclear	Unclear	Unclear	Low	Low	Low	Unclear
Bar Eli	Low	Unclear	Low	Low	Unclear	Low	Low	Low	Low
Battaglia	Low	Unclear	Unclear	Unclear	Unclear	Low	Low	Low	Unclear
Bedir	Low	Unclear	Unclear	Unclear	Unclear	Low	Low	Low	Unclear
Beilock	Low	Unclear	Unclear	Unclear	Unclear	Low	Low	Low	Unclear
Bjorkstrand	High	Unclear	Unclear	Unclear	Unclear	High	Low	Low	High
Blair	Low	Unclear	Low	Low	Low	Low	Low	Low	Low
Blakeslee	Low	Unclear	Unclear	Unclear	Unclear	Low	Low	Low	Unclear
Blumenstein	Low	Unclear	Unclear	Unclear	Unclear	Low	Low	Low	Unclear
Blumenstein (a)	Low	Unclear	Unclear	Unclear	Unclear	Low	Low	Low	Unclear
Blumenstein (b)	Low	Unclear	Unclear	Unclear	Unclear	Low	Low	Low	Unclear
Budnik-Przybylska	Low	Unclear	Unclear	Unclear	Unclear	Low	Unclear	Unclear	High
Callow	High	Unclear	Unclear	Unclear	Unclear	Unclear	Low	Low	High
Carter	Low	Unclear	Unclear	Unclear	Unclear	Low	Low	Low	Unclear
Christakou	Low	Unclear	Low	Unclear	Low	Low	Low	Low	Low
Chungath	Low	Unclear	Unclear	Unclear	Unclear	Low	Low	Low	Unclear
Coker	Low	Unclear	Unclear	Unclear	Unclear	Low	Low	Low	Unclear
Daneshfar	Low	Unclear	Unclear	Unclear	Unclear	Low	Low	Low	Unclear
Dello Iacono	Low	Unclear	Unclear	Unclear	Low	Low	Low	Low	Low
Dominique	Low	Unclear	Unclear	Unclear	Unclear	Low	Low	Low	Unclear
Fazel	Low	Unclear	Unclear	Unclear	Unclear	Low	Low	Low	Unclear

Author	Sequence generation	Allocation concealment	Blinding participants	Blinding personnel	Blinding outcome assessment	Incomplete outcome	Selective reporting	Other bias	Overall bias
Fekih	Low	Unclear	Unclear	Unclear	Unclear	Low	Low	Low	Unclear
Fekih(B)	Low	Unclear	Unclear	Unclear	Unclear	Low	Low	Low	Unclear
Fortes	Low	Low	Low	Low	Low	Low	Low	Low	Low
Fortes 2020	Low	Unclear	Unclear	Unclear	Unclear	Low	Low	Low	Unclear
Gmamdya	Low	Unclear	Unclear	Unclear	Unclear	Low	Low	Low	Unclear
Graham	Low	Low	Low	Low	Unclear	Low	Low	Low	Low
Gray	Low	Unclear	Unclear	Unclear	Unclear	Low	Low	Low	Unclear
Groslambert	Low	Unclear	Unclear	Unclear	Unclear	Low	Low	Low	Unclear
Grosso	Low	Unclear	Unclear	Unclear	Unclear	Low	Low	Low	Unclear
Guillot 2012	Low	Unclear	Unclear	Unclear	Unclear	Low	Low	Low	Unclear
Guillot 2015	Low	Unclear	Unclear	Unclear	Unclear	Low	Low	Low	Unclear
Hardy	High	Unclear	Unclear	Unclear	Unclear	Unclear	Low	Low	High
Hashmi	Low	Unclear	Unclear	Unclear	Unclear	Low	Low	Low	Unclear
Hidayat	Low	Low	Low	Unclear	Unclear	Low	Low	Low	Low
Howard	Low	Unclear	Unclear	Unclear	Unclear	Low	Low	Low	Unclear
Hut	Low	Unclear	Low	Low	Unclear	High	Low	Low	High
Itoh	Low	Unclear	Unclear	Unclear	Unclear	Low	Low	Low	Unclear
Jenny O	Low	Unclear	Low	Unclear	Unclear	Low	Low	Low	Low
Kanthack	Low	Unclear	Unclear	Unclear	Unclear	Low	Low	Low	Unclear
Khalaf	Low	Unclear	Unclear	Unclear	Unclear	Low	Low	Low	Unclear
Korim	Low	Unclear	Unclear	Unclear	Unclear	Low	Low	Low	Unclear
Lebon	Low	Unclear	Unclear	Unclear	Unclear	Low	Low	Low	Unclear
Lu	High	Unclear	Unclear	Unclear	Unclear	Low	Low	Low	Unclear
Malouff	Low	Unclear	Unclear	Unclear	Unclear	Low	Low	Low	Unclear

Author	Sequence generation	Allocation concealment	Blinding participants	Blinding personnel	Blinding outcome assessment	Incomplete outcome	Selective reporting	Other bias	Overall bias
Marshall	High	Unclear	Unclear	Unclear	Unclear	Low	Low	Low	High
McALENEY	Low	Unclear	Unclear	Unclear	Unclear	Low	Low	Low	Unclear
McNeill 2021	Low	Low	Low	Unclear	Unclear	Low	Low	Low	Low
Nagar	Low	Low	Low	Unclear	Unclear	Low	Low	Low	Low
Nassib	Low	Low	Low	Low	Low	Low	Low	Low	Low
Nassib 2017	Low	Unclear	Low	Unclear	Unclear	Low	Low	Low	Low
Neuman	Low	Unclear	Unclear	Unclear	Unclear	Low	Low	Low	Unclear
Nicolas	Low	Unclear	Unclear	Unclear	Unclear	Low	Low	Low	Unclear
Norouzi	Low	Unclear	Low	Unclear	Unclear	Low	Low	Low	Low
Olsson	Low	Unclear	Unclear	Unclear	Unclear	Low	Low	Low	Unclear
Plakoutsis	Low	Unclear	Low	Unclear	Unclear	Low	Low	Low	Low
Prasomsri	Low	Low	Low	Low	Unclear	Low	Low	Low	Low
Predebon	Low	Unclear	Unclear	Unclear	Unclear	Low	Low	Low	Unclear
Quinton	Low	Unclear	Unclear	Unclear	Unclear	Low	Low	Low	Unclear
Ramsey	Low	Unclear	Unclear	Unclear	Unclear	Low	Low	Low	Unclear
Rhodes	Low	Low	Low	Low	Low	Low	Low	Low	Low
Rienzo	Low	Unclear	Unclear	Unclear	Unclear	Low	Low	Low	Unclear
Robin	Low	Unclear	Unclear	Unclear	Unclear	Low	Low	Low	Unclear
Robin 2020	Low	Unclear	Unclear	Unclear	Unclear	Low	Low	Low	Unclear
Robin 2020B	Low	Unclear	Unclear	Unclear	Unclear	Low	Low	Low	Unclear
Robin 2022	Low	Unclear	Unclear	Unclear	Low	Low	Low	Low	Low
Robin 2022B	Low	Unclear	Unclear	Unclear	Unclear	Low	Low	Low	Unclear
Robin 2024	Low	Unclear	Unclear	Low	Unclear	Low	Low	Low	Low
Rodgers	Low	Unclear	Low	Low	Low	Low	Low	Low	Low
Rumeau	Low	Unclear	Low	Low	Unclear	Low	Low	Low	Low
Seif-Barghi	Low	Unclear	Low	Low	Unclear	Low	Low	Low	Low

Author	Sequence generation	Allocation concealment	Blinding participants	Blinding personnel	Blinding outcome assessment	Incomplete outcome	Selective reporting	Other bias	Overall bias
Selian	Low	Unclear	Unclear	Unclear	Unclear	Low	Low	Low	Unclear
Simonsmeier	Low	Unclear	Unclear	Unclear	Unclear	Low	Low	Low	Unclear
Slimani	Low	Unclear	Unclear	Unclear	Unclear	Low	Low	Low	Unclear
Smith	Low	Unclear	Unclear	Unclear	Unclear	Low	Low	Low	Unclear
Smith 2004	Low	Unclear	Unclear	Unclear	Unclear	Low	Low	Low	Unclear
Smith 2008	Low	Unclear	Unclear	Unclear	Unclear	Low	Low	Low	Unclear
Suedfeld	Low	Unclear	Unclear	Unclear	Unclear	Low	Low	Low	Unclear
Taylor	Low	Unclear	Unclear	Unclear	Unclear	Low	Low	Low	Unclear
Thelwell	Low	Unclear	Unclear	Unclear	Unclear	Low	Low	Low	Unclear
Wagaman	Low	Unclear	Unclear	Unclear	Unclear	Low	Low	Low	Unclear
Winter	Low	Unclear	Unclear	Unclear	Unclear	Low	Low	Low	Unclear
Wrisberg	Low	Unclear	Unclear	Unclear	Unclear	Low	Low	Low	Unclear
Yahya	Low	Low	Low	Low	Unclear	Low	Low	Low	Low
