# Supplementary material for: The Effects of Imagery Practice on Athletes’ Performance: A Multilevel Meta-Analysis with Systematic Review
Source: Behav Sci (Basel). 2025 May 16;15(5):685. doi: 10.3390/bs15050685 (PMC12109254; doi:10.3390/bs15050685)
Supplement: Supplementary file 1 [file behavsci-15-00685-s001.zip › Supplementary file S6 Moderation analysis.pdf]

Supplementary file S6: the Plots in Moderation Analysis

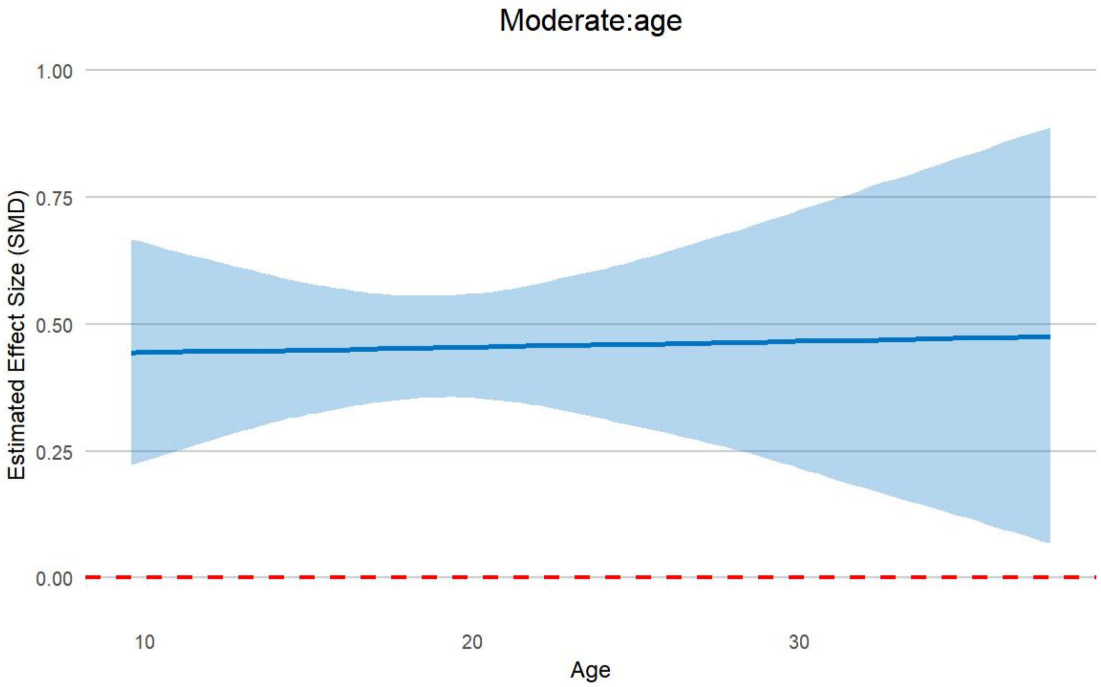

Figure 1 The regression plot by age

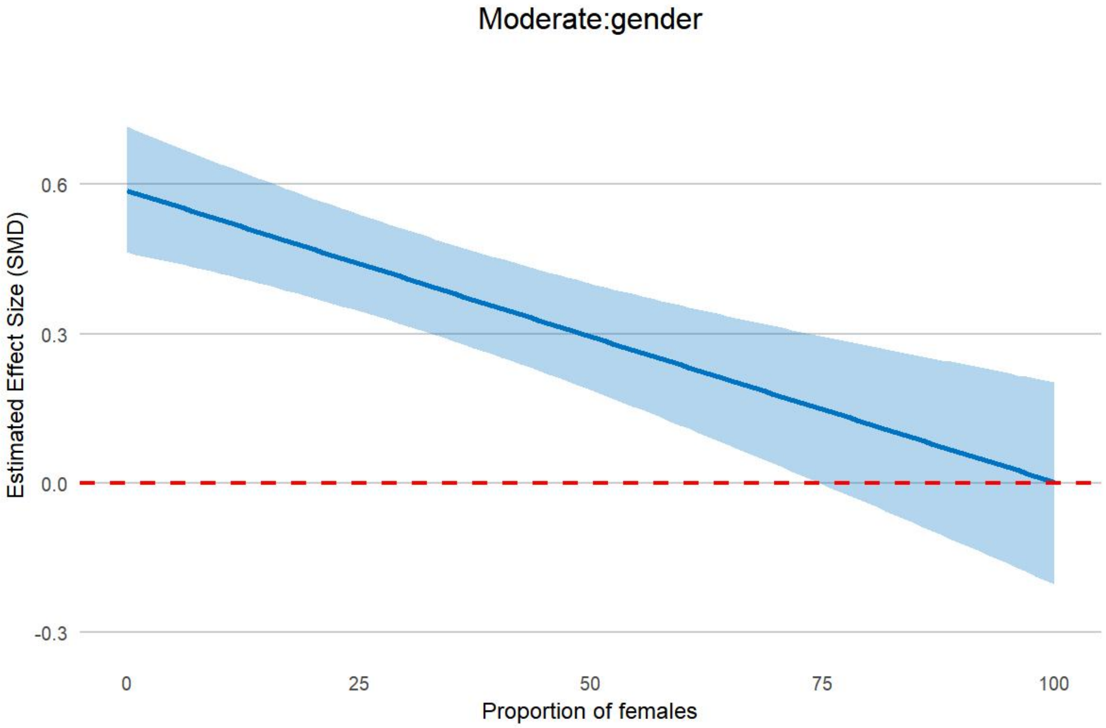

Figure 2 The regression plot by gender

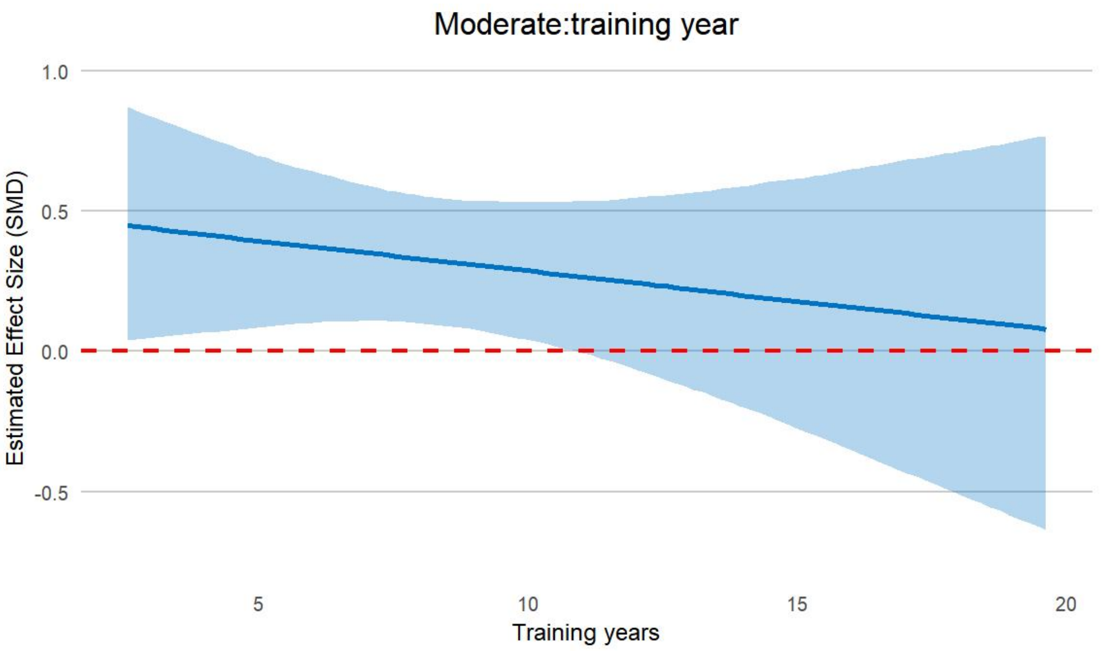

Figure 3 The regression plot by training years

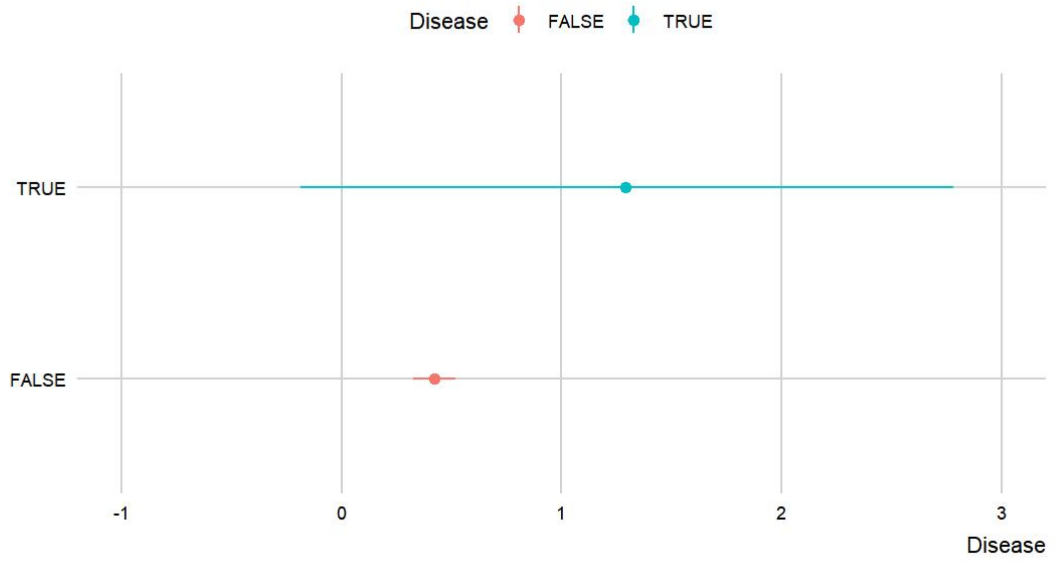

Figure 4 The moderation analysis by disease

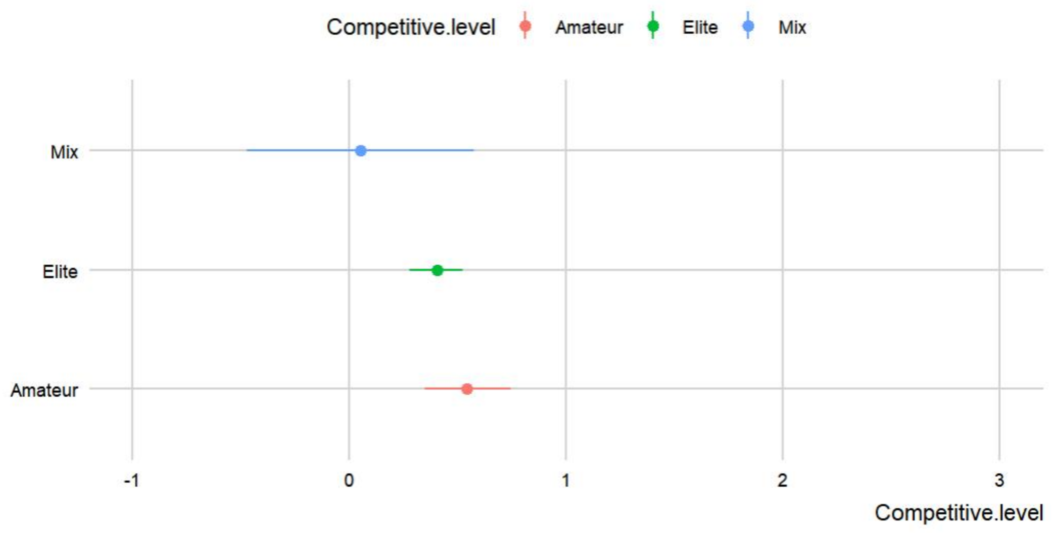

Figure 5 The moderation analysis by competitive level

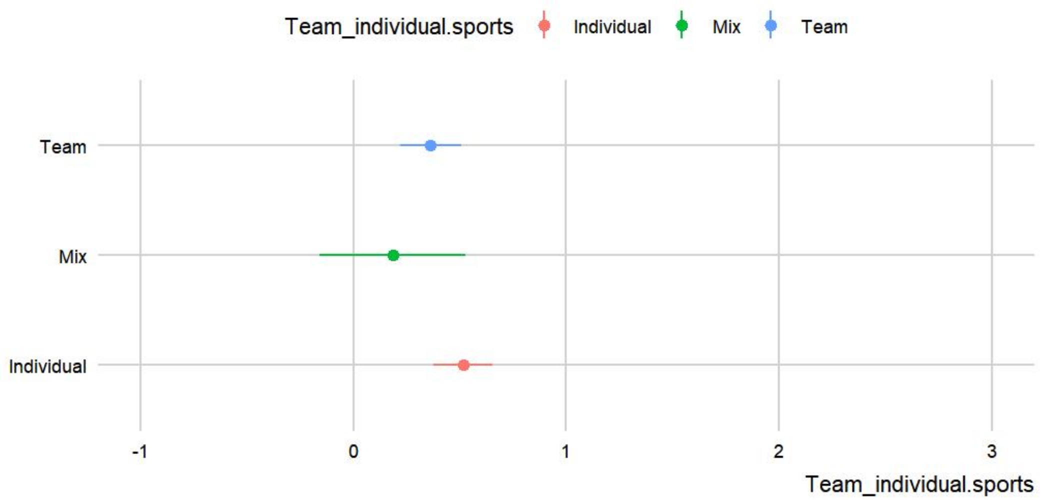

Figure 6 The moderation analysis by types of sports (team or individual sport)

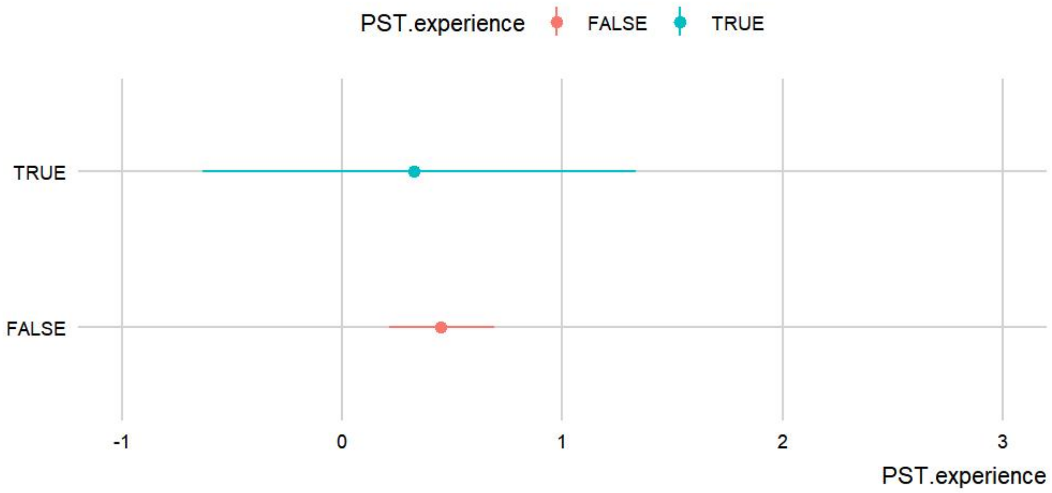

Figure 7 The moderation analysis by PST experience

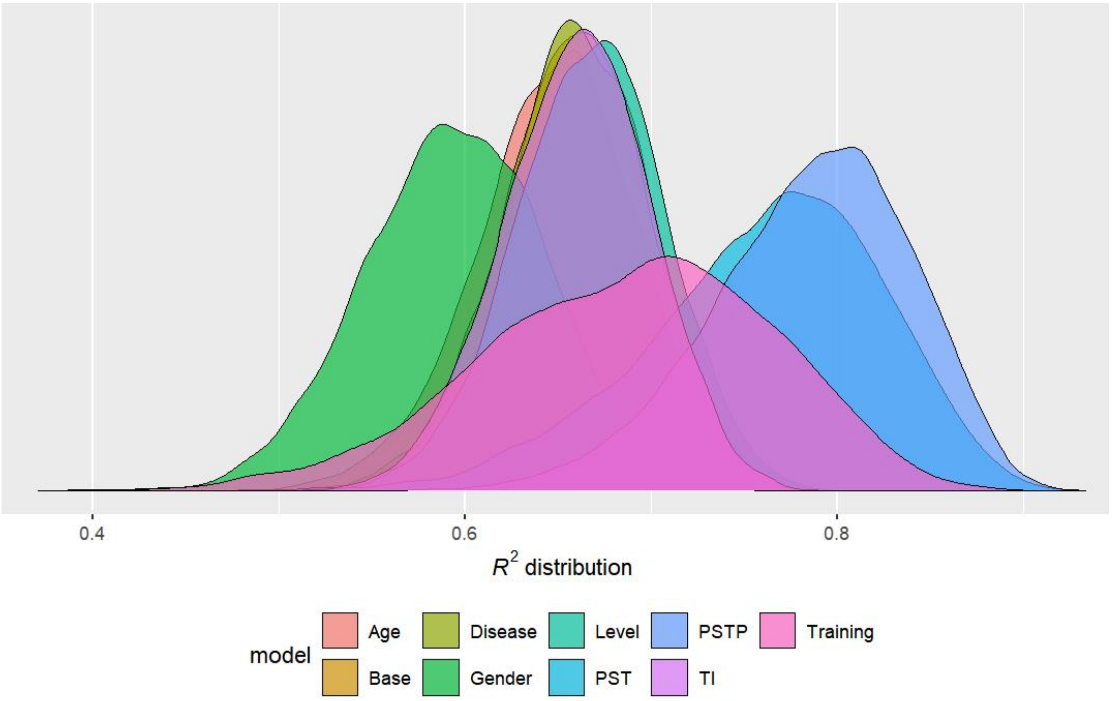

Figure 8 R^2 density plot in the moderation analysis of population characteristics

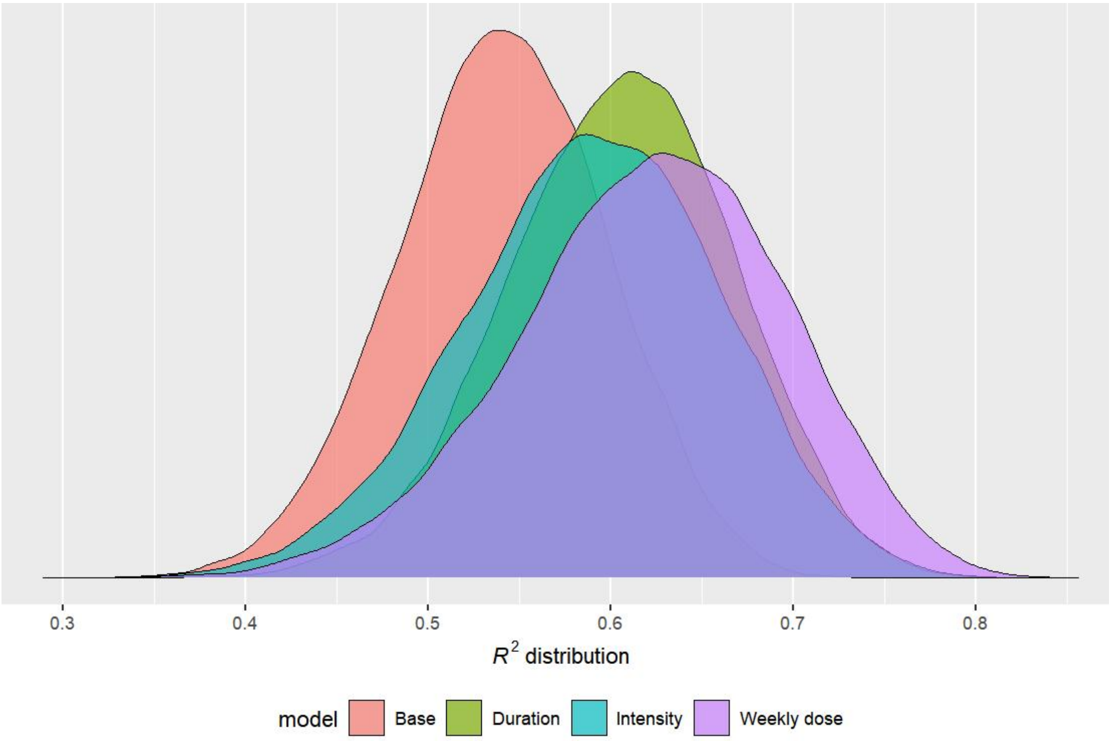

Figure 9 R^2 density plot in the moderation analysis of imagery dosage
