# Supplementary figures and images for: The Effects of Imagery Practice on Athletes’ Performance: A Multilevel Meta-Analysis with Systematic Review
Source: Behav Sci (Basel). 2025 May 16;15(5):685. doi: 10.3390/bs15050685 (PMC12109254; doi:10.3390/bs15050685)

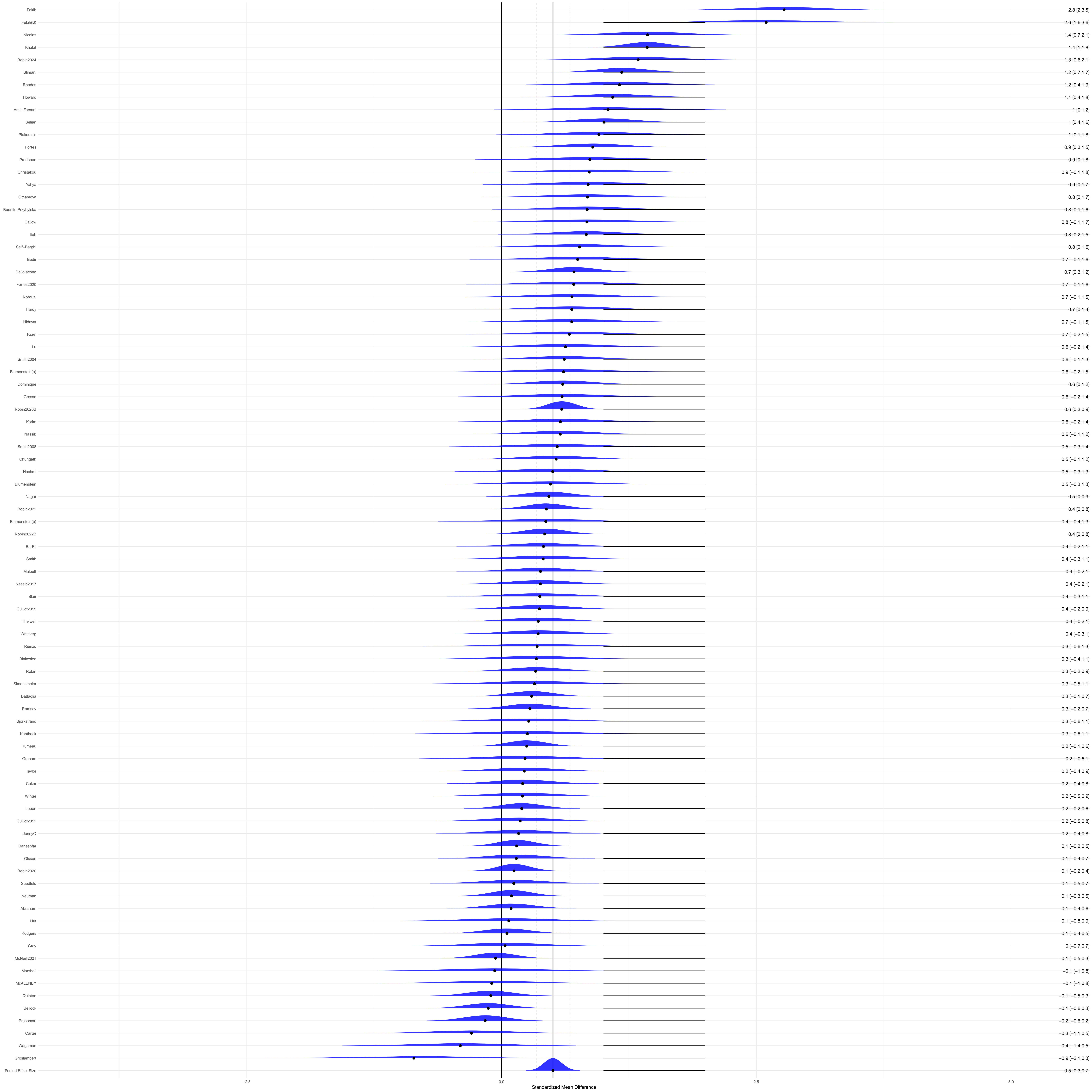

Supplement: Supplementary file 1 [file behavsci-15-00685-s001.zip › Supplementary file S5 forest plot in zero model.pdf]
